# Antibiofilm Activity of Manuka Honey in Combination with Antibiotics

**DOI:** 10.1155/2014/795281

**Published:** 2014-02-26

**Authors:** Michelle E. M. Campeau, Robin Patel

**Affiliations:** ^1^Division of Clinical Microbiology, Department of Laboratory Medicine and Pathology, Mayo Clinic, 200 First Street S.W., Rochester, MN 55905, USA; ^2^Mayo High School, 1420 11th Avenue S.E., Rochester, MN 55904, USA; ^3^Division of Infectious Disease, Department of Medicine, Mayo Clinic, 200 First Streer S.W., Rochester, MN 55905, USA

## Abstract

We assessed the *in vitro* activity of Manuka honey against biofilm bacteria in combination with antibiotics and visualized the effect of Manuka honey on bacterial biofilms using scanning electron microscopy. The fractional biofilm eradication concentration (∑FBEC) index for vancomycin plus Manuka honey against *S. aureus* IDRL-4284 biofilms was 0.34, indicating a synergistic interaction. The ∑FBEC index for gentamicin plus Manuka honey against *P. aeruginosa* PAO1 biofilms was 0.78–0.82, indicating an additive interaction. Scanning electron microscopy of *S. aureus* IDRL-4284 and *P. aeruginosa* PAO1 biofilms exposed to Manuka honey and artificial honey containing the same sugar composition as Manuka honey showed that the former had more pronounced effects than the latter on both *S. aureus* and *P. aeruginosa* biofilms. Visualized effects included distorted cell morphologies for both bacteria and a decrease in *P. aeruginosa* extracellular matrix. In conclusion, Manuka honey has a synergistic interaction with vancomycin against *S. aureus* biofilms and an additive interaction with gentamicin against *P. aeruginosa* biofilms.

## 1. Introduction

Medicinal honeys are registered for topical application in several countries and are formulated in tubes, into ointments and gels, and made into wound dressings. A common medicinal honey is Manuka honey, from the Manuka bush (*Leptospermum scoparium*), indigenous to New Zealand and Australia. Manuka honey has a high sugar content (high osmolarity) and low pH. Nonperoxide components are predominantly responsible for its antimicrobial activity [1, 2]. It contains the antibacterial methylglyoxal [3, 4]; although it also contains bee defensin-1, bee defensin-1 is modified by methylglyoxal, abrogating its antibacterial activity [5].

Manuka honey has *in vitro* activity against planktonic bacteria. It also possesses *in vitro* antibiofilm activity [6–13]. Given the topical uses of Manuka honey, its antibiofilm activity is clinically important, especially since most traditional antibiotics lack activity against biofilms. Preliminary studies demonstrate activity of Manuka honey against biofilms in humans [6, 14].

In medical practice, Manuka honey might be delivered in combination with traditional antibiotics, with both being administered topically or with Manuka honey being administered topically and the traditional antibiotic(s) being administered systemically. Therefore, it is useful to understand the interaction between Manuka honey and conventional antibiotics. Jenkins and Cooper reported a synergistic interaction between oxacillin and Manuka honey against a single strain of *Staphylococcus aureus* grown in the planktonic state [15]. They followed up their report by testing 15 antibiotics with Manuka honey against planktonic *S. aureus* and *Pseudomonas aeruginosa* using a variety of methods; five antibiotics exhibited improved activity with Manuka honey [16]. Aside from these studies, there has been little evaluation of the combination of Manuka honey with traditional antibiotics, and this strategy has not, to the best of our knowledge, been evaluated against bacterial biofilms.

Our goals were to evaluate the activity of Manuka honey with antibiotics against biofilm bacteria and to visualize effects of Manuka compared to artificial honey on biofilms using electron microscopy.

## 2. Materials and Methods

### 2.1. Bacterial Strains

*S. aureus* IDRL-4284 (a clinical isolate) and *P. aeruginosa* PAO1 were studied.

### 2.2. Manuka and Artificial Honey

Active Manuka Honey UMF 16+ (Manuka Honey USA LLC, Orlando, FL http://www.manukahoneyusa.com) and artificial honey were studied. Artificial honey was prepared according to Cooper et al. by dissolving 3 g sucrose, 15 g maltose, 81 g D-fructose, and 67 g D-glucose in 34 mL sterile water [17] and irradiating the solution with 4 kGy gamma irradiation. Manuka honey was used as purchased. For testing described below, honey was dissolved in cation-adjusted Mueller Hinton broth (CAMHB) and confirmed to be sterile by plating an aliquot onto a sheep blood agar plate which was subsequently incubated overnight at 37°C.

### 2.3. Treatment of Established Biofilms with Combinations of Manuka Honey and Antibiotics

The effect of Manuka honey and vancomycin alone and in combination against *S. aureus*, or Manuka honey and gentamicin alone and in combination against *P. aeruginosa*, was assessed using a modification of a previously-described assay for treatment of established biofilms [18]. Vancomycin was chosen for study against *S. aureus* because it is commonly used for serious staphylococcal infections and has activity against most *S. aureus* isolates, regardless of methicillin susceptibility. Gentamicin, an aminoglycoside, was chosen for study against *P. aeruginosa*; aminoglycosides may be administered topically or systemically for the treatment of *P. aeruginosa* infection. Antibiotic solutions were prepared from stock solutions of 8 mg of vancomycin or gentamicin in 4 mL of CAMHB; concentrations tested were 0, 1, 2.5, 5, 10, 15, 20, 25, 50, 75, 100, and 500 *μ*g/mL. For *S. aureus*, concentrations of Manuka honey tested alone and in combination with vancomycin were 0, 1, 2, 3, 4, 5, 7.5, and 10% (vol/vol) in CAMHB; for *P. aeruginosa*, concentrations of Manuka honey tested alone and in combination with gentamicin were 0, 10, 15, 17.5, 20, 22.5, 25, and 30% (vol/vol) in CAMHB. Five colonies of each bacterium were placed in 10 mL of trypticase soy broth (TSB), vortexed, and incubated overnight at 37°C. Then, they were adjusted to a turbidity of 1.0 McFarland, diluted 1 : 50 in TSB, and vortexed. 150 *μ*L aliquots of bacterial solution was placed into wells of a 96-well microtiter well plate with a pegged lid and incubated overnight at 37°C. The next day, the pegged lids were placed into plates containing 200 *μ*L Manuka honey and/or vancomycin or gentamicin in CAMHB and incubated overnight at 37°C. The following day, pegged lids were placed in microtiter well plates containing sterile water for 30 seconds (to rinse off the honey and/or antibiotic) and then transferred to a microtiter well plate containing 200 *μ*L of CAMHB per well. The OD_600_ of the plate containing the honey and/or antibiotic was measured using a spectrophotometer, and the minimum inhibitory concentration (MIC) estimated as the lowest concentration of honey or antibiotic that resulted in an OD_600_ < 0.2 for *S. aureus* and <0.4 for *P. aeruginosa* (because of the higher concentrations of Manuka honey studied). The CAMHB plates were incubated overnight at 37°C. The following day, the pegged lids were removed and the OD_600_ was measured using a spectrophotometer. The minimum biofilm eradication concentration (MBEC) was defined as the lowest concentration of honey or antibiotic to result in an OD_600_ < 0.200.

The fractional biofilm eradication concentration (FBEC) of vancomycin or gentamicin in combination with Manuka honey was calculated as follows [19]:

(1) $$\begin{array}{c} \frac{\text{MBEC}\;\text{of}\;\text{antibiotic}\;\text{in}\;\text{the}\;\text{presence}\;\text{of}\;\text{Manuka}\;\text{honey}}{\text{MBEC}\;\text{of}\;\text{antibiotic}\;\text{alone}}. \end{array}$$

The FBEC of Manuka honey was calculated as follows:

(2) $$\begin{array}{c} \frac{\text{MBEC}\;\text{of}\;\text{Manuka}\;\text{honey}\;\text{in}\;\text{the}\;\text{presence}\;\text{of}\;\text{antibiotic}}{\text{MBEC}\;\text{of}\;\text{Manuka}\;\text{honey}}. \end{array}$$

The two FBEC values were added to give the ∑FBEC index. An ∑FBEC index of ≤0.5 indicates a synergistic effect, >0.5 to 1 indicates an additive effect, >1 to <2 indicates indifference, and ≥2 indicates antagonism.

### 2.4. Electron Microscopy of Biofilms Exposed to Manuka and Artificial Honey

*S. aureus *and *P. aeruginosa *biofilms on Teflon discs were exposed to Manuka and artificial honey and imaged using scanning electron microscopy. For *P. aeruginosa*, Manuka honey concentrations of 15, 20, 25, and 30% and an artificial honey concentration of 50% (vol/vol) in CAMHB were studied. For *S. aureus*, Manuka honey concentrations of 1.5, 3, 5, 7.5, and 10% and an artificial honey concentration of 50% (vol/vol) in CAMHB were studied. The higher artificial honey concentrations were selected because prior experiments had shown artificial honey to have MBEC values >35% for both *S. aureus* IDRL-4284 and *P. aeruginosa* PAO1, higher than the respective values for Manuka honey (data not shown). For each honey concentration studied, two discs were prepared, one for quantitative culture and another for electron microscopy. 12.5 mm diameter discs were cut from a 1 mm thick Teflon sheet using a punch biopsy instrument and hammer. After marking each with a black dot on one side for orientation purposes, they were autoclaved. Five colonies of each bacterium were placed in 45 mL of TSB, vortexed, and incubated overnight at 37°C. After incubation, the solutions were adjusted to a turbidity of 1.0 McFarland, diluted 1 : 50 in TSB, and vortexed. 1 mL aliquots of bacterial solution was placed into wells of a 24-well flat bottomed microtiter plate. A sterile disc was placed into each well using tweezers, with the dot not showing. The plates were incubated overnight at 37°C. The next day, after being dipped into sterile water, the discs were moved into a new 24 well plate containing honey solutions in CAMHB and incubated overnight at 37°C.

The following day, the discs for electron microscopy were placed in 2.5 mL of Trump's solution (4% formaldehyde and 1% glutaraldehyde in a phosphate buffer, pH 7.3) and refrigerated. The discs for quantitative culture were dipped in sterile water for 1 minute and placed in 2.5 mL TSB in a test tube, vortexed for 30 seconds, and bath sonicated for 5 minutes. Quantitative cultures were performed by plating five serial ten-fold dilutions in TSB onto sheep blood agar plates which were incubated overnight. The concentration of bacteria was calculated to determine which discs to examine using electron microscopy, and the respective discs in Trump's solution were submitted for scanning electron microscopy at the Mayo Clinic scanning electron microscopy core facility. Following critical-point drying and gold-palladium sputter coating, disks were imaged by cold-field emission scanning electron microscopy using a Hitachi S-4700 electron microscope (Hitachi Ltd., Tokyo, Japan).

## 3. Results and Discussion

### 3.1. Treatment of Established Biofilms with Combinations of Manuka Honey and Antibiotics

The MIC and the MBEC of Manuka honey against *S. aureus* IDRL-4284 were 2 and 3%, respectively; the MIC and the MBEC of vancomycin were ≤1 and 100 *μ*g/mL, respectively. The MIC and the MBEC of Manuka honey against *P. aeruginosa* PAO1 were 20 and 30%, respectively; the MIC and the MBEC of gentamicin were 10 and 500 *μ*g/mL, respectively. Similar to our findings, Cooper et al. used an agar dilution method to show that the MIC of Manuka honey against *S. aureus* was 3% [17], a finding Jenkins et al. recently showed extends to vancomycin-intermediate *S. aureus* [20]. Maddocks et al., however, reported higher MIC values, ranging from 6 to 20% using a broth microdilution method and four *S. aureus* isolates [13]. Cooper et al. used an agar dilution method to show that the mean MIC of Manuka honey against *P. aeruginosa* was 8% [17], and Mullai and Menon reported a Manuka honey MIC of 10% against *P. aeruginosa* ATCC 27853 [21]; these values are slightly lower than found herein, possibly due to strain or methodology differences. Using a broth microdilution method, Maddocks et al. recently reported Manuka honey MIC values of 10 and 30% for two strains of *P. aeruginosa*, supporting strain-dependent variability [13]. There have been limited studies evaluating the effect of Manuka honey on *S. aureus* and *P. aeruginosa* biofilms. Alandejani et al. studied *S. aureus* and *P. aeruginosa* biofilms but only evaluated 50% Manuka (and Sidr) honey [7]. They reported that 50% Sidr and Manuka honey killed 68 and 73%, respectively, of the biofilms of 22 *S. aureus* strains tested and 91 and 91%, respectively, of 11 *P. aeruginosa* strains tested. We studied lower and likely more clinically relevant concentrations of Manuka honey. Majtan et al. have recently shown that methylglyoxal accounts for antibiofilm activity of Manuka honey [12].

We showed that subinhibitory Manuka honey concentrations increased vancomycin's activity against *S. aureus* biofilms and had an additive effect with gentamicin against *P. aeruginosa* biofilms (Table 1). Specifically, the fractional biofilm eradication concentration (∑FBEC) index for the combination of vancomycin and Manuka honey against *S. aureus *biofilms was 0.34, indicating a synergistic interaction. The ∑FBEC index for the combination of gentamicin and Manuka honey against *P. aeruginosa* biofilms was 0.78–0.82, indicating an additive interaction.

Although it has been suggested that Manuka honey may improve the activity of conventional antibiotics against planktonic bacteria [15, 16, 22], as far as we know, no one has previously reported the antibiofilm activity of Manuka honey in combination with antibiotics. This is clinically relevant because medicinal honey is used topically in an environment where biofilms play a role, and conventional antibiotics may also be topically or systemically administered in cases of biofilm-associated infections managed with topical Manuka honey. It is possible that by combining Manuka honey and traditional antibiotics, emergence and spread of traditional antibiotic resistance may be impeded. Since traditional antibiotics and Manuka honey have different modes of action [23], one would be unlikely to select for cross-resistance to the other. Reassuringly, Cooper et al. have shown that the risk of emergence of resistance to Manuka honey itself is low [24].

### 3.2. Electron Microscopy of Biofilms Exposed to Manuka and Artificial Honey

Teflon discs with *S. aureus* biofilm exposed to 0 or 10% Manuka honey, or 50% artificial honey, which yielded 7.6, 7.2, and 7.4 log_10_ colony forming units (cfu)/disc, respectively, and discs with *P. aeruginosa* biofilm exposed to 0 or 20% Manuka honey, or 50% artificial honey, which yielded >7.6, 3.8 and 5.4 log_10_ cfu/disc, respectively, were subjected to scanning electron microscopy.

**Figure 1:**
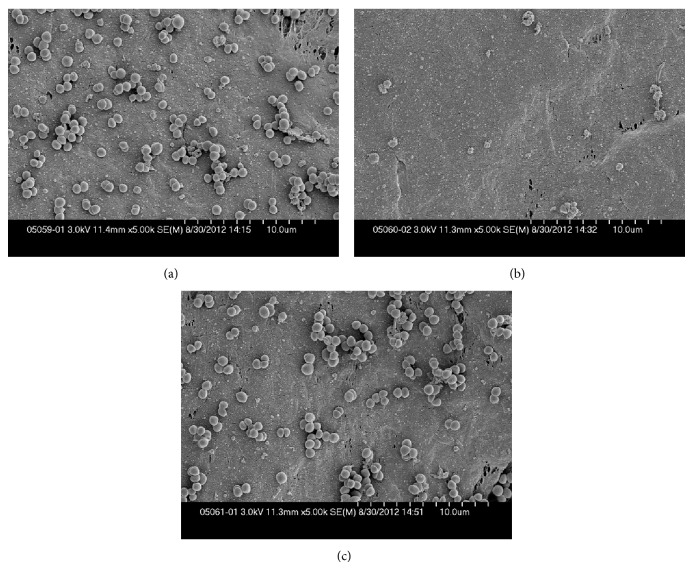
Scanning electron microscopy of *S. aureus* biofilms (scale bar, 1 *μ*m)—(a) control; (b) 10% Manuka honey; (c) 50% artificial honey (representative images). The control biofilm appeared as numerous cells, all round spheres; those dividing retained their spherical shape. When treated with Manuka honey, the cell surfaces appeared rough and the cells were no longer spherical. The number of cells on the disc was dramatically decreased. The artificial honey appeared to have little effect on the cells; they appeared similar to the control cells in appearance and density.

**Figure 2:**
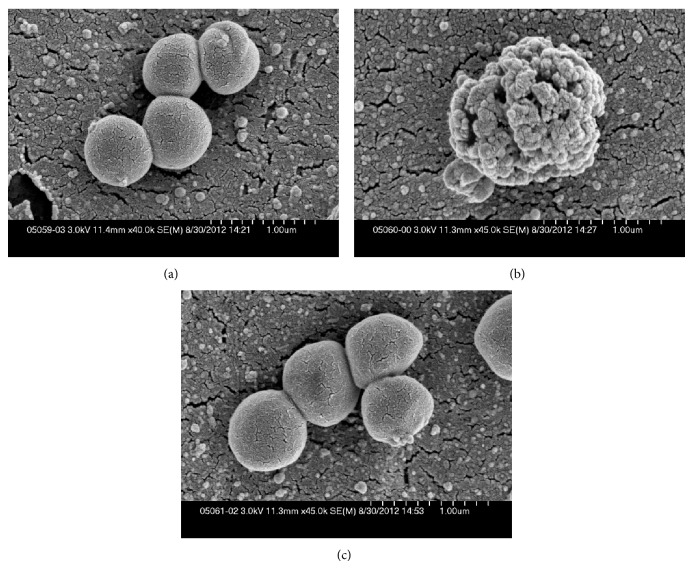
Scanning electron microscopy of *S. aureus* biofilms (scale bar, 0.1 *μ*m)—(a) control; (b) 10% Manuka honey; (c) 50% artificial honey (representative images). In these close-up images, the differences seen in Figure 1 are emphasized. The Manuka honey-exposed cell had many holes and crevices in its surface, whereas the control cells and those exposed to artificial honey had a smooth exterior. The Manuka honey-exposed cells also appeared larger than the control or artificial honey-exposed cells.

Images of *S. aureus* biofilms are shown in Figures 1 and 2. Manuka honey-exposed cells had roughened outer surfaces, were slightly enlarged, and had numerous surface holes and crevices. There was a noticeable difference in cell density compared to control biofilms, mirroring results of quantitative culture. The artificial honey-exposed cells had a similar overall appearance to control cells.

**Figure 3:**
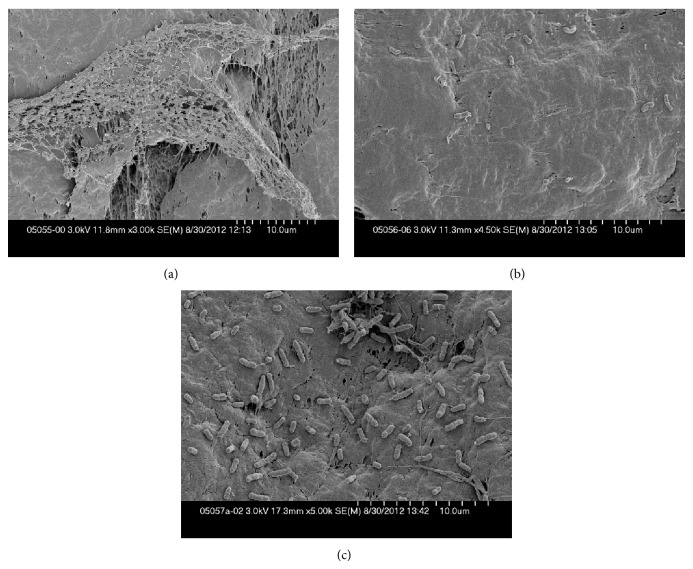
Scanning electron microscopy of *P. aeruginosa *biofilms (scale bar, 1 *μ*m)—(a) control; (b) 20% Manuka honey; (c) 50% artificial honey (representative images). The control image showed hundreds of bacterial cells connected by a substantial amount of extracellular matrix which appeared stringy and covered most of the cells. The Manuka honey-exposed bacterial cells, in contrast, were spread apart with little apparent matrix. The cells appeared curved and distorted, and the cell density was markedly decreased. The artificial honey-exposed cells had a few connecting strands, but were mainly separate from one another. They less densely covered the disc than the control cells but more densely covered the disc than did the Manuka honey-exposed cells.

**Figure 4:**
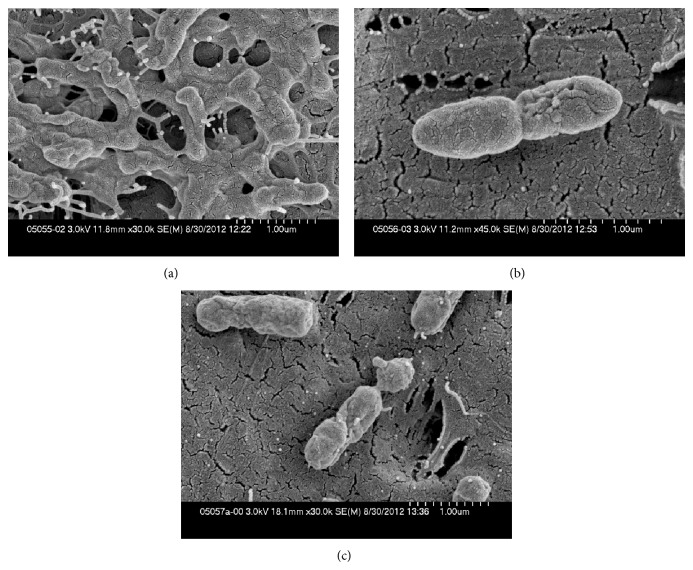
Scanning electron microscopy of *P. aeruginosa* biofilms (scale bar, 0.1 *μ*m)—(a) control; (b) 20% Manuka honey; (c) 50% artificial honey (representative images). In these higher magnification views, the matrix was seen only in the control images. The Manuka honey-exposed cell had multiple furrows. The Manuca honey-exposed cell appeared to be trying to divide. The artificial honey-exposed cells appeared similarly furrowed and also appeared to be trying to divide. The control biofilm was composed of many cells; the honey-exposed biofilms consisted of fewer cells.

Images of *P. aeruginosa* biofilms are shown in Figures 3 and 4. Manuka honey exposure was associated with less extracellular matrix compared to control cells. The cells were bent and had indentations in their surfaces compared with the relatively smooth control cells. When treated with artificial honey, there was also a decrease in extracellular matrix; the cells more densely covered the disc than did the Manuka honey-exposed cells. The artificial honey appeared to have more activity at the same concentration against *P. aeruginosa* than *S. aureus* biofilms, as evidenced by both the scanning electron microscopy and quantitative cultures.

Few investigators have visualized effects of Manuka honey on bacteria using electron microscopy, and to the best of our knowledge, none have done so with biofilm bacteria. Henriques et al. performed scanning electron microscopy on planktonic *P. aeruginosa* cells exposed to Manuka honey and observed loss of structural integrity and changes in cell shape, including shorter cells, distortion, and surface defects [25]. Lu et al. described both longer and shorter planktonic *P. aeruginosa* cells in response to Manuka honey exposure, depending on growth phase [2]. Jenkins et al. reported that Manuka honey-exposed planktonic *S. aureus* cells were enlarged and had more septa [26], whereas Lu et al. reported shorter planktonic cells [2].

Together, our results show that Manuka honey has visually appreciable activity against established *S. aureus* and *P. aeruginosa* biofilms, that subinhibitory Manuka honey concentrations increase vancomycin's activity against *S. aureus* biofilms, and that Manuka honey has an additive interaction with gentamicin against *P. aeruginosa* biofilms.

## 4. Conclusions

Manuka honey has *in vitro* antibiofilm activity. We demonstrated that subinhibitory concentrations of Manuka honey increase the killing activity of vancomycin against *S. aureus* biofilms and that Manuka honey has an additive interaction with gentamicin against *P. aeruginosa* biofilms. This interaction may be clinically useful in medical practices where topical Manuka honey might be delivered in combination with conventional antibiotics. We also used scanning electron microscopy to visualize the effect of Manuka honey on *S. aureus* and *P. aeruginosa* biofilms; distorted morphologies of both species were seen, along with a decreased amount of *P. aeruginosa* biofilm extracellular matrix.

## Figures and Tables

**Table 1:** ∑FBEC indices.

	MBEC of antibiotic		MBEC of Manuka honey		∑FBEC index
	Alone	With Manuka honey			
*P. aeruginosa *PAO1 (gentamicin)	500 µg/mL	100 µg/mL with 17.5% Manuka honey	30%	17.5% with 100 µg/mL gentamicin	0.78
		75 µg/mL with 20% Manuka honey		20% with 75 µg/mL gentamicin	0.82

*S. aureus* IDRL-4284 (vancomycin)	100 µg/mL	1 µg/mL with 1% Manuka honey	3%	1% with 1 µg/mL vancomycin	0.34
